# Effect of CaSO_4_ Incorporation on Pore Structure and Drying Shrinkage of Alkali-Activated Binders

**DOI:** 10.3390/ma12101673

**Published:** 2019-05-23

**Authors:** Hyeongmin Son, Sol Moi Park, Joon Ho Seo, Haeng Ki Lee

**Affiliations:** Department of Civil and Environmental Engineering, Korea Advanced Institute of Science and Technology, 291 Daehak-ro, Yuseong-gu, Daejeon 34141, Korea; nemilhm@kaist.ac.kr (H.S.); solmoi.park@kaist.ac.kr (S.M.P.); junhoo11@kaist.ac.kr (J.H.S.)

**Keywords:** slag, fly ash, CaSO_4_, pore structure, drying shrinkage

## Abstract

This present study investigates the effects of CaSO_4_ incorporation on the pore structure and drying shrinkage of alkali-activated slag and fly ash. The slag and fly ash were activated at a 5:5 ratio by weighing with a sodium silicate. Thereafter, 0%, 5%, 10%, and 15% of CaSO_4_ were incorporated to investigate the changes in phase formation and internal pore structure. X-Ray Diffraction (XRD), thermogravimetry (TG)/derivative thermogravimetry (DTG), mercury intrusion porosimetry (MIP), nuclear magnetic resonance (NMR), and drying shrinkage tests were carried out to find the correlation between the pore structure and drying shrinkage of the specimens. The results showed that CaSO_4_ incorporation increased the formation of thenardite, and these phase changes affected the pore structure of the activated fly ash and slag. The increase in the CaSO_4_ content increased the pore distribution in the mesopore. As a result, the capillary tension and drying shrinkage decreased.

## 1. Introduction

A significant amount of CO_2_ is produced during cement production, which has substantially impacted global warming and air pollution [1]. Approximately 7% of the world’s CO_2_ emissions are due to Portland cement production. For example, 1 ton of CO_2_ is produced per ton of Portland cement [2]. As interest in the environment has increased, numerous studies have focused on developing binders that can replace cement [3,4,5,6]. Alkali-activated materials (AAM), such as blast furnace slag and fly ash, which can reduce carbon dioxide emissions, have recently been receiving attention as binders in place of conventional cement [7,8]. Slag and fly ash are currently used as representative binders that provide silicate (SiO_2_) and aluminate (Al_2_O_3_) as an aluminosilicate precursor instead of cement. Therefore, AAM is used for various fields such as transportation, industry, residential, mining, and well cementing [9,10], as well as for special purposes, such as radioactive waste disposal and toxic metal immobilization [11,12,13].

AAM is a composite synthesized by using inorganic material rich in silicate (SiO_2_) and aluminate (Al_2_O_3_) with an alkaline solution [14]. Numerous studies on the types of activators [15,16] and the types of binders [17,18,19,20,21,22] used in manufacturing AAM are currently in progress. MOH and M_2_O are frequently used as alkali activators, where M represents Na or K [15]. Slag and fly ash have been primarily used as the binders of AAM, and the reaction products have differed due to the chemical composition of both materials.

Calcium-rich slag-based AAM produces C-S-H gels with low Ca/Si ratios [23]. In contrast, the other products generated are hydrotalcite [24]. Slag-based AAM is structurally different compared to Portland cement, which mainly consists of SiQ^1^ and SiQ^2^ in a linear finite chain in the C-S-H gel. However, slag-based AAM has a long chain structure comprised of SiQ^2^ or SiQ^2^ (1Al) [23]. 

Fly ash, which has a high silica and aluminum content, can form an amorphous aluminosilicate gel as the primary product of the reaction [24]. This aluminosilicate gel has a three-dimensional network structure [25], which differs from a linear finite chain. In particular, the AAM that uses fly ash rich in silicon and aluminum shows a higher chemical resistance and thermal conductivity [26].

Recently, research on blended AAM with calcium-rich slag and fly ash that has high silicate and aluminum content has focused on utilizing these single binders [1,27]. It has been confirmed that a binder mixed with fly ash and slag has a high sulfuric acid resistance compared to only alkali activated slag due to the formation of more C-S-H gels and improvements in the distribution of the pore size [28,29]. When the two materials are mixed, the products of the reactions are complicated and differ from those produced when the fly ash and slag are used in isolation [23,26,30]. 

A significant number of studies have been conducted to improve the mechanical performance and control the setting time by incorporating an admixture into the AAM. Park et al. demonstrated that incorporating gypsum in the GGBFS activated by clinker-free CaO can lead to an ettringite formation that produces a positive effect on strength enhancement [31]. Although the addition of phosphoric acid in sodium silicate-based alkali-activated slag pastes has increased the setting time and decreased the initial compressive strength, the incorporation of gypsum increased the compressive strength and decreased the setting time [32]. In the case of fly ash with gypsum, a significant strength enhancement was observed in comparison with the strength of the unreacted fly ash after three months [33]. The formation of ettringite was observed as the incorporation of gypsum increased in high volume fly ash cement [34].

Aimin et al. showed that ettringite phase formation increased with an additional amount of gypsum in high volume fly ash cement [35]. Moreover, the addition of gypsum accelerated the geopolymerization of the bottom ash and activation of alumina leaching due to the presence of sulfate ions [36]. Neto et al. proved that incorporating sodium silicate and silica into a blast furnace slag (BFS) can result in additional ettringite formation as well as decreased shrinkage [37]. However, there is a lack of detailed studies on the pore structure when blended AAM is mixed with slag and fly ash that incorporate an admixture [38,39].

This study investigated the effect of CaSO_4_ incorporation on the pore structure and drying shrinkage of alkali-activated slag and fly ash (AASF). It also demonstrated the influence of anhydrite on AAFS when slag and fly ash were used as main binders with the alkaline activator. CaSO_4_ from 0 to 15% quantities in the weight ratio was incorporated into a slag/fly ash binder activated by a sodium silicate. X-ray diffraction (XRD), thermogravimetrics (TGs), and nuclear magnetic resonance (NMR) were used to characterize the hydration products. Mercury intrusion porosimetry (MIP) was conducted to examine the pore structure of the hardened alkali-activated slag/fly ash paste.

## 2. Materials and Methods 

### 2.1. Materials and Specimens Preparation

The chemical compositions of the Class F fly ash and BFS used in the present study are shown in Table 1. The mortar specimens were fabricated with a 0.4:1:2 weight ratio of water:binder (fly ash and slag):fine aggregate. The fly ash and slag were actively mixed at a 5:5 ratio with 8% sodium silicate. A sodium silicate as an alkali-activator (SiO_2_ = 47.0%, Na_2_O = 50.0%, and bulk density = 1.2 g/cm^3^) of a powder type was used to synthesize the paste and mortar. The effect of incorporating CaSO_4_ on the pore structure of the AAFS was investigated by replacing a portion of the fly ash/slag with varying quantities of CaSO_4_ (0, 5, 10, and 15% relative to the weight of the binder). Table 2 shows the mixing proportions utilized in the present study. 

The specimens underwent the following procedure. First, dry mixtures of fly ash/slag, sand, and CaSO_4_ were stirred to ensure homogeneity for five minutes. Water was then added to the mixture and stirred for an additional five minutes for homogeneous blending with the alkaline activator. Subsequently, the mixture was poured into a 50 × 50 × 50 mm^3^ mold for a compressive strength test and into a 30 × 30 × 600 mm^3^ mold for the dry shrinkage test. To determine the compressive strength and paste specimens, the mortar was sealed in a plastic wrap to prevent moisture from evaporating. The mortar was also cured at 20 °C. The specimens for the drying shrinkage test were installed one day after casting. The specimens for the drying shrinkage measurements were de-molded one day after casting and were cured at a room temperature of 20 °C and 50% relative humidity.

### 2.2. Test Methods

XRD, TG, MIP, and NMR analyses were conducted to investigate the effect of incorporating CaSO_4_ on the pore structure and phases of AAFS. The specimens for the analyses were immersed in acetone and dehydrated for 48 h to remove any capillary water. They were then subsequently crushed and made to pass through a 100-µm sieve. 

The XRD analysis was conducted at a scan speed of 0.5 °/min using the Rigaku D/MAX-2500 (Rigaku, Tokyo, Japan) with Cu-Kα radiation and a scan range of 5° to 65°. The TG/DTG analysis was conducted using TGA/DSC1/1600LF (Columbus, OH, USA) in N2 gas heated at a rate of 10 °C/min. 

MIP analysis was conducted using the ASTM D4284-07 method on an Autopore VI machine made by Micromeritics Instruments Corporation (Norcross, GA, USA). The contact angles and the surface tension of mercury were 130° and 485 dynes, respectively, for MIP analysis. 

Solid-state ^29^Si MAS NMR spectral analysis was conducted at 79.51 MHz using an HX-CP MAS probe (Bluker, Billerica, MA, USA) and a 4-mm zirconia rotor with a Teflon spacer at a spinning speed of 11.0 kHz. A pulse width of 30° and 2.2 μs with a relaxation delay of 22 s was employed for the ^29^Si MAS NMR analysis. The chemical shifts were referenced to tetrakis (trimethylsiyl) silane at −135.5 ppm. 

Solid-state ^27^Al MAS NMR spectra were collected at 156.3 MHz using an HX-CPMAS probe and a 2.5-mm outer diameter, as well as a low-Al background zirconia rotor at a spinning speed of 22.0 kHz. A pulse width of 30° and 1.8 μs with a relaxation delay of 2 s was employed. The chemical shifts were referenced to an external specimen of aqueous AlCl_3_ at 0 ppm for ^27^Al MAS NMR analysis. 

The drying shrinkage was measured in accordance with ASTM 490 using a dial gauge. The first day’s measurements were taken hourly, the measurements for the rest of the first week were taken twice a day, and from the second week onwards, the measurements were taken once a day.

## 3. Experimental Results and Discussion

### 3.1. Shrinkage of Mortar Specimens

**Figure 1:**
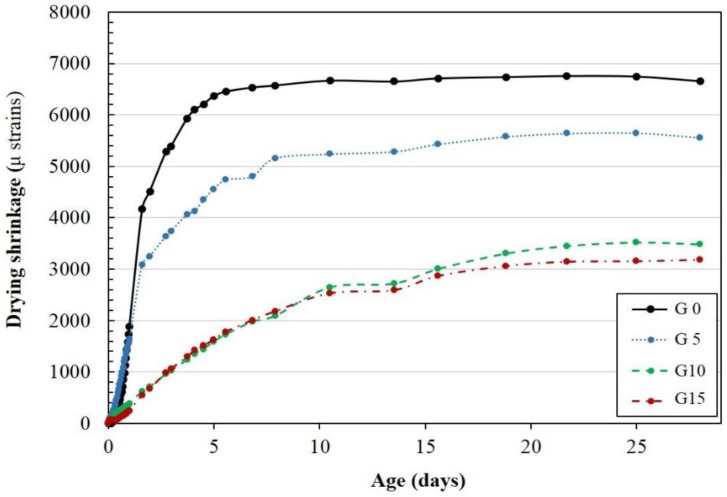
Drying shrinkage of the specimens.

**Figure 2:**
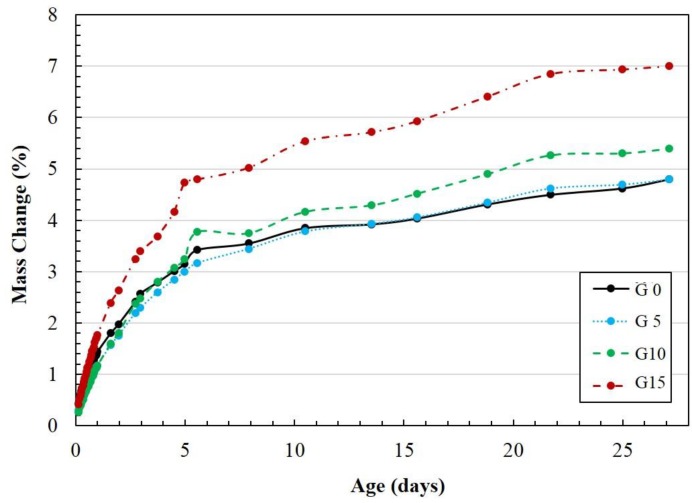
Mass change of the specimens.

Drying shrinkage and mass change rates of specimens incorporating CaSO_4_ content are shown in Figure 1 and Figure 2, respectively. When 0% CaSO_4_ was incorporated, the highest drying shrinkage was observed in the paste, while G15 displayed the lowest drying shrinkage. The drying shrinkage difference between G10 and G15 was insignificant. In addition, the rate of growth of the drying shrinkage increased with a decrease in CaSO_4_ content. It was observed that the CaSO_4_ content affected the degree of shrinkage and the rate of shrinkage by changing the amount of water that evaporated. Therefore, a higher content of CaSO_4_ resulted in a lower mass loss due to a higher amount and faster water evaporation rate. This phenomenon is closely related to the pore size distribution [37]. This result means that the initial water evaporation occurs in the major capillary pores, and as the curing progresses, water evaporation in the intermediate micropores occurs [37]. The mass reduction of G15 by water evaporation was the largest, but the drying shrinkage in G15 was the smallest. It is reasonable that the rapid evaporation from the mesopore region has a greater effect than the evaporation from macropores [38].

**Figure 3:**
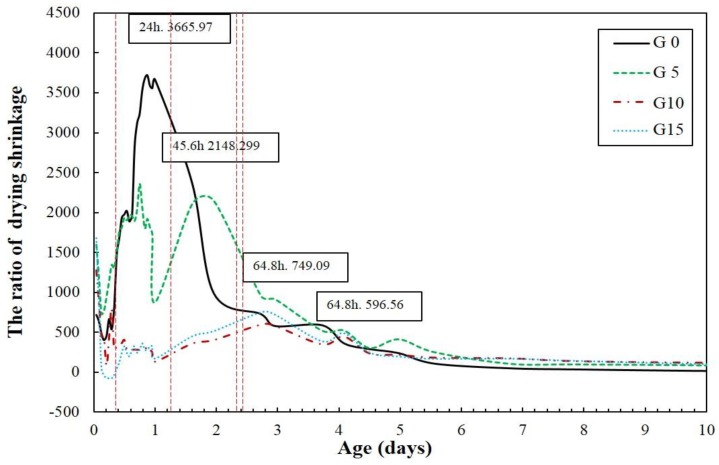
The ratio of drying shrinkage of the specimens.

The drying shrinkage rate from incorporating CaSO_4_ is shown in Figure 3. It can be observed that as the CaSO_4_ quantity increases, the ratio of drying shrinkage decreases. In addition, the highest peak drying shrinkage is delayed. The peak times at which the maximum drying shrinkages occur are 24, 45.6, 64.8, and 67 h. The results of drying shrinkage rates with different CaSO_4_ quantities show the existence of an initial shrinkage peak followed by a secondary peak. These peaks are closely related to the microstructure development and the hydration kinetics of each specimen [37]. The first peak is caused by the evaporation of free water after de-molding, whereas the second peak is related to the evaporation of water from the mesopores [37]. 

### 3.2. Effect of CaSO4 Incorporation on the Hydration Product of Paste Specimens

**Figure 4:**
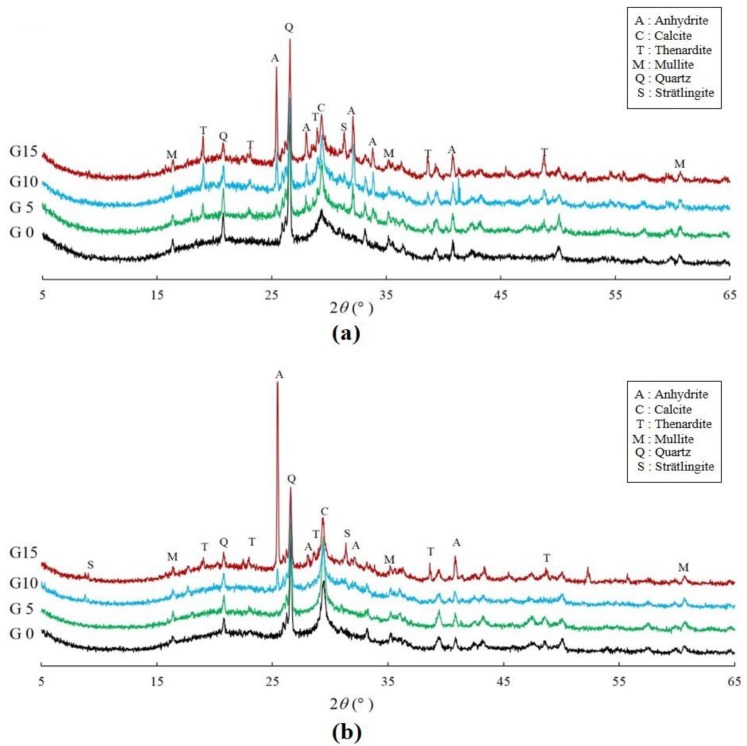
X-ray diffraction (XRD) patterns of the specimens on the (**a**) 7th day and (**b**) 28th day.

**Figure 5:**
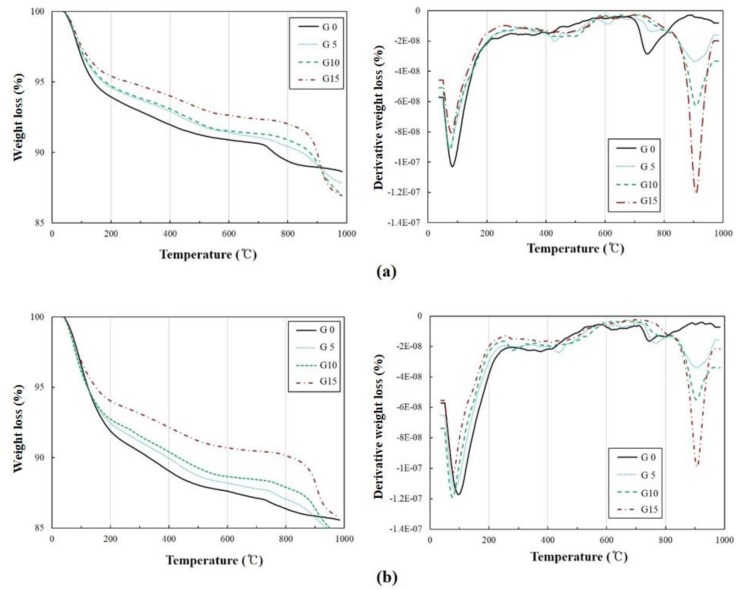
Thermogravimetric (TG)/DTG results on the (**a**) 7th day and (**b**) 28th day.

The results of XRD and DTG on the 7th and 28th days are shown in Figure 4 and Figure 5, respectively. The peaks of mullite and quartz were observed due to the presence of the unreacted fly ash. Previous studies have reported that when slag is used as a binder, ettringite is produced as a reaction of the sulfate [31]. Although the generation of ettringite was not significant in all the specimens, a peak of thenardite was observed, and its intensity increased with an increase in the CaSO_4_ content. The DTG results showed a primary peak between 50 to 100 °C and around 900 °C when the decomposition of the C-S-H gel and thenardite occurred [40,41,42]. The mass reduction in the C-S-H gel was less as the amount of CaSO_4_ incorporated was increased on the 7th day. Thenardite, which decomposes at around 900 °C, had a tendency to increase when the amount of CaSO_4_ incorporated was increased. The TG and DTG results showed a difference in the amount of C-S-H gel produced when the CaSO_4_ quantities were mixed. On the 7th day, as the CaSO_4_ content increased, the mass reduction between 100 and 200 °C also increased [43]. The results of the specimens’ TG and DTG on the 28th day show that the C-S-H gel formation decreased with an increase in the CaSO_4_ content.

A significant difference in the peak intensity of the anhydrite (CaSO_4_) was observed. Although the peak intensity of the G0’s anhydrite was insignificant, as the amount of CaSO_4_ increased, the peak intensity of the anhydrite was noticeable. The phase changes that interpreted the effect of CaSO_4_ on the pore structure and the drying shrinkage of the alkali-activated fly ash of the slag in the present study differ from those in the previous studies.

**Figure 6:**
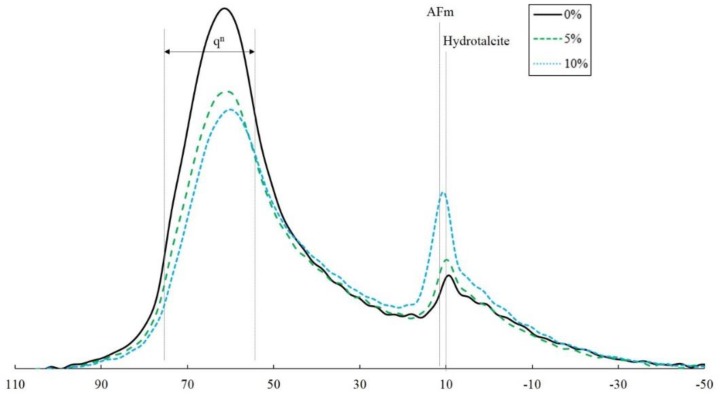
^27^Al nuclear magnetic resonance (NMR) of the specimens on the 28th day.

The ^27^Al MAS NMR results of each specimen on the 28th day showed the same results as the XRD in ettringite formation (Figure 6). The q^2^ and q^3^ peaks from 80 ppm to 60 ppm increased with an increase in CaSO_4_ incorporation. The resonance at 8 and 12 ppm denotes the presence of hydrotalcite and monosulfate, respectively. An increase in the incorporated CaSO_4_ quantity resulted in an increase in the peak near 10 ppm, where the peak indicates the presence of monosulfate. Therefore, an increase in the CaSO_4_ content can be consumed to form monosulfate and hydrotalcite with the addition of the Al gel in the C-S-H gel. The NMR did not show the formation of ettringite, and the greatest difference due to CaSO_4_ incorporation was confined to the formation of thenardite and anhydrite. The primary reason for no ettringite formation could be that any ettringite produced was transformed into mono sulfate due to an insufficient SO_4_ supply [44,45]. A further hypothesis can be made that ettringite was initially formed at an early stage and was then transformed into mono sulfate due to an inadequate SO_4_ supply [46,47]. The initially formed ettringite acted as a barrier that suppressed the hydration reaction of the binder. Consequently, the amount of unreacted slag increased with an increase in the amount of CaSO_4_ and the small amount of C-S-H gel produced.

### 3.3. Effect of CaSO_4_ Incorporation on Amorphous Gel of Paste Specimens

**Figure 7:**
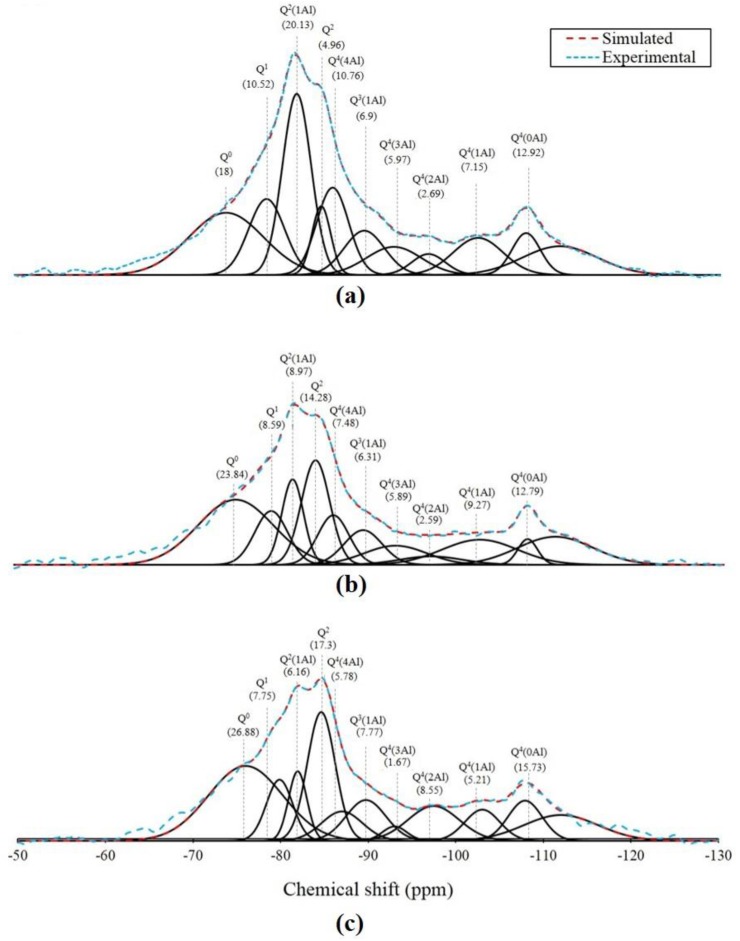
^29^Si NMR of the specimens on the 28th day, (**a**) 0% CaSO_4_, (**b**) 5% CaSO_4_, (**c**) 10% CaSO_4_.

The ^29^Si NMR spectra of the CaSO_4_ that incorporated AAFS on the 28th day of curing are shown in Figure 7a–c, which represent the specimens’ incorporation of 0%, 5%, and 10% CaSO_4_, respectively. The spectra show that the predominant reaction product was C-A-S-H, which can be = attributable to the Q^1^, Q^2^(1Al), Q^2^, and Q^3^(1Al) sites with a significantly lower intensity of Q^4^(nAl) (n = 1, 2, 3, and 4) sites featured on the spectra. The summation of the relative areas corresponding to the C-A-S-H and N-A-S-H products of the G0 specimen was 35.61% and 32.54%, respectively. This indicates that the degree of reaction for the fly ash did not progress as much as that of the slag. However, the relative areas of both the C-A-S-H and N-A-S-H decreased with an increase in the CaSO_4_ content, thereby implying that the CaSO_4_ inhibits the reaction of AAFS. The summation of the reaction product (i.e., the entire relative area of C-A-S-H and N-A-S-H) for the G0 specimen was 69.08%, whereas that of the G5 and G10 specimens were 63.38% and 60.19%, respectively. This phenomenon is partially reflected by the TG/DTG results that indicate a reduction of C-A-S-H with an increase in the CaSO_4_ content.

### 3.4. The Correlation Between the Pore Structure and Drying Shrinkage of Paste Specimens

**Figure 8:**
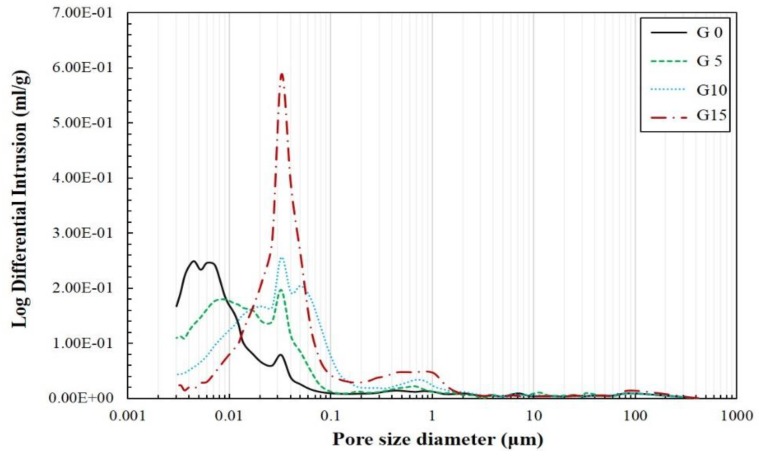
Pore size distribution on the 28th day.

Figure 8 shows the pore size distribution results of each specimen with respect to the CaSO_4_ content on the 28th day as revealed by the MIP test. Their pore characteristics are tabulated in Table 3. The MIP peak intensities vary depending on the amount of CaSO_4_ mixed. The smaller the CaSO_4_ content, the more the pore distribution in the micropore was below 1.25 nm, whereas, as the CaSO_4_ content increased, the pore distribution was relatively prominent in the mesopore (1.25–25 nm). The increase in the CaSO_4_ content resulted in an increase in the peak in the macropore (25–5000 nm). The highest peak intensity was obtained when the amount of CaSO_4_ was 15%. Previous studies have found that the drying shrinkage is closely related to the volume of the mesopore [48]. A higher pore volume in the mesopore range is associated with a larger capillary tension by the water meniscus produced in the capillary pores of pastes, which is caused by a higher level of drying shrinkage [49]. The increase in the CaSO_4_ content causes a growth of the pores in the mesopore. Moreover, the decrease in the drying shrinkage is similar to the results of the previous studies [48].

The capillary pores are composed of the mesopore and micropore [50]. The micropore is composed of gel pores from the calcium aluminate silicate hydrate gel. Although an increase in the amount of CaSO_4_ resulted in the pores increasing in micro size, the pores in the mesopore decreased. When CaSO_4_ was not incorporated, the micropore that occupied the pores inside the C-S-H gel accounted for a significantly large part. However, the micropore size decreased with an increase in the CaSO_4_ content, as depicted by the TG/DTG results. Therefore, CaSO_4_ has an adverse effect on the formation of the C-S-H gel.

The changes in pores due to the incorporation of CaSO_4_ can affect the drying shrinkage of a hardened paste. A significant number of studies have been conducted on the correlation between the existing pore distribution and the drying shrinkage [51,52,53]. Also, the C-S-H gel and pore size distribution have a critical effect on the drying shrinkage [54]. Based on these results, it can be assumed that the incorporation of CaSO_4_ reduced the amount of C-S-H gel in AAM, resulting in a decrease in the micropores, which contributed to a higher drying shrinkage. A large pore size distribution in the mesopore resulted in larger capillary tension. Consequently, the drying shrinkage was reduced [55]. In addition, in the analysis of the ^29^Si NMR results, it was observed that the C-A-S-H gel and N-A-S-H gel reduced when CaSO_4_ was incorporated, which means the decrease of capillary tension resulting in drying shrinkage.

## 4. Concluding Remarks

The present study investigated the effect of CaSO_4_ incorporation on the phase and the internal voids of AAFS, as well as the effects on drying shrinkage. The CaSO_4_ was incorporated in weight ratios of 0%, 5%, 10%, and 15% in sodium silicate-activated fly ash/slag. The primary results of this study can be summarized as follows:

1. An increase in CaSO_4_ incorporation resulted in an increase in the unreacted slag, and hence, the amount of C-S-H gel decreased.
2. An increase in the CaSO_4_ content increased the number of voids distributed in the mesopore size, thereby decreasing the capillary tension and reducing the drying shrinkage.
3. The increase in the quantity of CaSO_4_ increased the formation of thenardite due to the combination of SO_3_ and Na ions. Consequently, it can be inferred that the number of SO_3_ ions needed for ettringite formation decreased.
4. The CaSO_4_ incorporation in AASF contributed to the increase in mesopore size, which may have caused a decrease in drying shrinkage by reducing the capillary tension.

This study shows that the incorporation of CaSO_4_ may have reduced the drying shrinkage by changing the pore structure of the alkali-activated fly ash/slag specimens. Future studies that investigate the effect of CaSO_4_ incorporation on the strength of AASF need to be carried out to determine the correlation between pore structure and mechanical properties.

## Figures and Tables

**Table 1:** Chemical composition of fly ash and slag used in this study.

(wt %)	SiO_2_	Al_2_O_3_	Fe_2_O_3_	CaO	MgO	P_2_O_5_	TiO_2_	K_2_O	SO_3_	LOI ^a^
Fly ash	50.0	21.0	10.0	4.8	1.3	1.5	1.5	1.4	1.0	2.71
Slag	32.4	11.5	0.6	47.7	3.0	0.6	0.5	0.5	2.7	0.29

^a^ Loss on ignition.

**Table 2:** Mix composition of the specimens.

Specimen Code	Slag	Fly Ash	Gypsum	Water	Sand	W/B ^a^
G0	0.5	0.5	0	0.4	2	0.40
G5	0.475	0.475	0.05	0.4	2	0.40
G10	0.45	0.45	0.1	0.4	2	0.40
G15	0.425	0.425	0.15	0.4	2	0.40

^a^ Water/binder ratio.

**Table 3:** Pore characteristics on the 28th day.

Specimen Code	Porosity (vol %)	Total Pore Area (m^2^/g)	Average Pore Diameter (nm)
G0	33.35	103.791	8.1
G5	33.74	77.919	11.9
G10	38.17	50.620	20.4
G15	43.05	40.908	29.7
